# One-year mortality and Periprosthetic infection rates after Total knee Arthroplasty in Cancer patients: a population-based cohort study

**DOI:** 10.1186/s12885-018-4329-2

**Published:** 2018-06-04

**Authors:** Feng-Chen Kao, Yao-Chun Hsu, Pang-Yu Lai, Chang-Bi Wang, Yuan-Kun Tu, Wen-Kang Chen

**Affiliations:** 1 Department of Orthopaedics, E-Da Hospital, Kaohsiung, Taiwan; 2 School of Medicine, I-Shou University, Kaohsiung, Taiwan; 3 School of Medicine, Fu-Jen Catholic University, New Taipei, Taiwan; 4 Department of Internal Medicine, E-Da Hospital, Kaohsiung, Taiwan; 5 Graduate Institute of Clinical Medical Science, China Medical University, Taichung, Taiwan; 6 Department of Oncology, E-Da Hospital, Kaohsiung, Taiwan; 7 Graduate Institute of Public Health, China Medical University, Taichung, Taiwan; 8 Department of Statistics, National Taipei University, Taipei, Taiwan; 9 Department of Applied Cosmetology, National Tainan Junior College of Nursing, Tainan, Taiwan; 10 Division of Gastroenterology and Hepatology, Fu-Jen Catholic University Hospital, New Taipei, Taiwan

**Keywords:** Total knee arthroplasty, Cancer survivors, Survival rate, revision rate

## Abstract

**Background:**

Knowledge on periprosthetic infection and mortality rate following total knee arthroplasty (TKA) is essential for justifying this treatment in patients with cancer; however, relevant data from population-based studies are lacking. Therefore, we examined 1-year periprosthetic infection, mortality, and 5-year relative survival rates in cancer patients who underwent TKA.

**Methods:**

This is a population-based cohort study based on analysis of the Taiwan National Health Insurance Research Database. We enrolled a total of 2294 cancer patients and 131,849 patients without cancer (control group) who underwent TKA between January 1, 1997, and December 31, 2011. All patients were followed until death, infection, withdrawal from the National Health Insurance, or December 31, 2012.

**Results:**

The periprosthetic knee joint infection rate in cancer patients (1.73%) was not significantly higher than that in the control group (1.87%). However, the 1-year mortality rate was significantly higher (*p* < 0.05) in the cancer group (4.10%) than in the control group (1.66%). The overall 5-year survival rate was 93.10% as compared with those without cancers.

**Conclusion:**

Low periprosthetic knee joint infection rates and high 5-year relative survival rates indicate the feasibility of TKA in cancer patients. However, the surgeon should take into account a higher mortality rate in the first year following TKA.

## Background

The prevalence of cancer continues to increase because of the aging population, sedentary lifestyles, increased obesity, and improved survival amongst cancer patients. In 2011, the Health Promotion Administration Ministry of Health and Welfare in Taiwan reported that there were approximately 100,000 new cancer patients every year in this country, translating to an annual incidence of about 0.41% [1]. The median age of patients diagnosed with cancer was 62 years [1]. The survival rates for some cancers have increased because of advances in treatment as well as in cancer prevention and screening [2]. There were more than 10 million cancer survivors in the United States in 2007 [2].

The majority of the cancer survivors are elderly people, who are generally affected by osteoarthritis (OA), one of the most common disorders affecting the musculoskeletal system of this population, resulting in physical deficiencies and poor quality of life [3, 4]. In severe cases of OA [5, 6], total knee arthroplasty (TKA) is an effective treatment option for relieving knee pain and restoring joint function. However, the operation of TKA in the elderly may lead to adverse effects that include longer hospital stay, higher incidences of surgical complications, and higher mortality rates [7–15]. Hence, elderly cancer patients may find it difficult to decide whether to undergo TKA and might be apprehensive about spending the rest of their lives with a TKA prosthesis.

In this 10-year population-based retrospective study, we used the Taiwan National Health Insurance Research Database (NHIRD) to examine the one-year postoperative surgical infection rate and survival rate in cancer patients who underwent TKA. In addition, we used the demographic and medical data of these patients to evaluate the risk factors for mortality after TKA.

## Methods

### Data source

Through the National Health Insurance (NHI) program, established in 1995, the Taiwan Department of Health covers the health care of 22.9 million residents of Taiwan, which is > 99% of the total population. The medical claims from 1997 to 2012 of these insurants are encrypted and released for research by the Taiwan National Health Research Institutes as the NHIRD. The Department of Health and the NHI Administration Bureau of Taiwan ensure the completeness and accuracy of the NHIRD.

The study data were obtained from the NHIRD. All patients included in the analysis were followed for outcome identification by using the International Classification of Disease, Ninth Revision, Clinical Modification, codes until the end of 2012. Because the NHIRD contains encrypted and deidentified data, this study was exempted from a full ethics review. This study was approved by the institutional Review Board of E-Da Hospital, Taiwan (EMRP-103-011; EMRP-103-012) and the Taiwan NHRI (NHIRD-103-116).

### Definition of study groups and outcomes

**Fig. 1:**
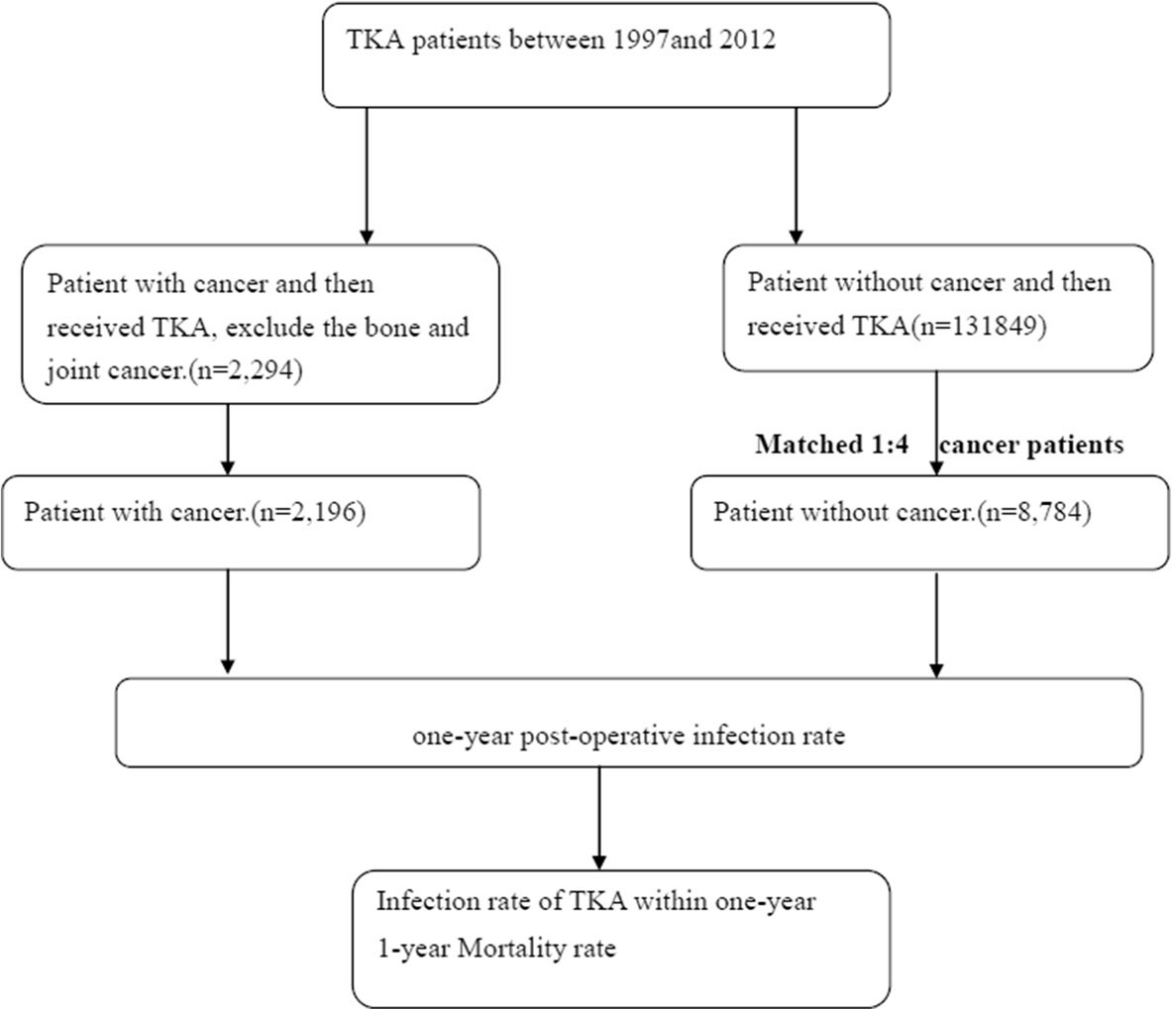
Study flow chart

Patients diagnosed with cancer before receiving TKA between January 1, 1997, and December 31, 2011, were identified from the NHIRD and included in the TKA group. Patients with musculoskeletal cancers were not enrolled. We also excluded those whose cancer was diagnosed more than 5 years before undergoing TKA, because the cancer status might be considered as having been cured in some of these patients. Their inclusion would be inappropriate given that our analysis aimed to inform the decision making on TKA surgery in cancer patients, instead of those who had cured a cancer. Taking into account that data in the NHIRD could not ascertain whether a cancer was cured, we restricted the eligibility to emphasize our study population. The control group comprised patients without cancer who underwent TKA. One-year postoperative infection rates and mortality rates in the two groups were evaluated and compared (Fig. 1). The one-year postoperative mortality rates was calculated from the analysis of survival rate of patients (with/without cancer) who underwent TKA. Periprosthetic joint infection was defined as the occurrence of surgically treated osteomyelitis or septic arthritis in the vicinity of the joint implants. Surgical interventions included debridement, prosthesis removal, or resection arthroplasty (Appendix). Conditions existing prior to TKA were classified as comorbidities according to Charlson’s score [16]. Among the cancer patients, the number with and without metastasis and those who had received chemotherapy at the time of TKA were also calculated (Appendix). All patients were followed until death, withdrawal from the NHI program, or the end of the study period (December 31, 2012). Figure 1 illustrates the study flow chart.

Subcohorts were defined to evaluate the overall 5-year survival rate of cancer patients who underwent TKA. In addition, the 1- and 5-year relative survival rates were calculated [17, 18].

### Statistical analysis

Pearson’s chi-square test and Fisher Yates continuity correction were used to compare categorical variables. Student’s t test was used for continuous ones. Traditional survival analysis only considers one event at a time (e.g., death or infection). Thus, certain events may be overlooked, and the resulting risk values may be overestimated. Therefore, these results should not be directly interpreted and applied in clinical settings. Our study considered mortality and the competing risk survival analysis using the Fine and Gray regression model [19] to calculate subdistribution hazards, and *p*-values were determined using Gray’s test. *P* < 0.05 was considered significant. All statistical tests and subdistribution hazard ratio (sHR) calculations were performed using Statistical Analysis Software, Version 9.4 (SAS Institute, Cary, NC, USA).

## Results

### Baseline characteristics of the patients

**Fig. 2:**
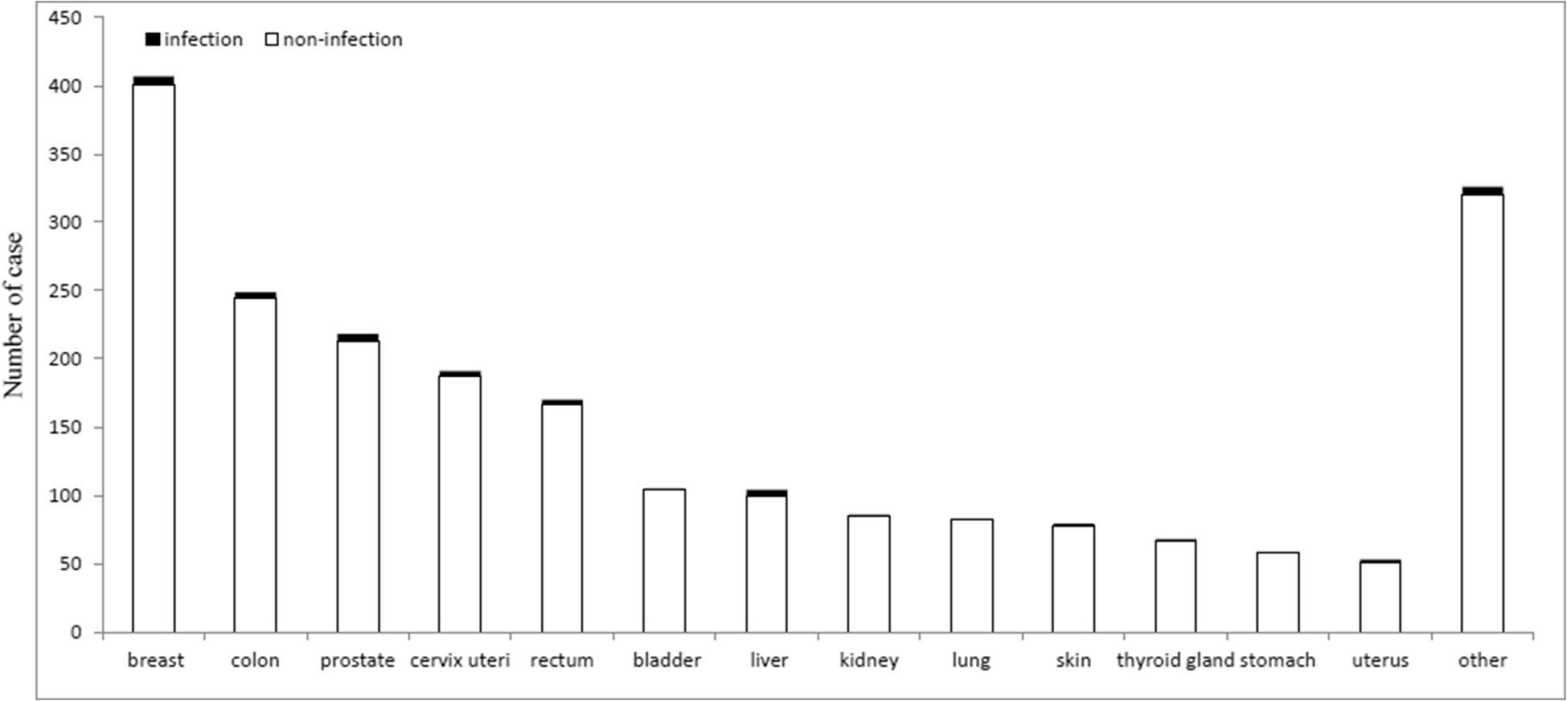
Distribution of different types of cancer and periprosthetic knee joint infectio

A total of 2294 cancer patients who underwent TKA were identified and included in the TKA group, whereas the control group comprised 131,849 patients. Score matching for gender and Charlson’s comorbidity index yielded 2196 patients in the TKA group and 8784 in the control group. Among these 10,980 patients, 3355 (30.56%) were male and 7625 (69.44%) female, and 10,236 (93.22%) patients were older than 60 years. The baseline characteristics and comorbidities of all patients are listed in Table 1. Among the cancer patients, 7 (0.32%) cases had metastatic diseases and 708 (32.34%) of them ever received chemotherapy when TKA was performed. The most common cancers were breast, colon, prostate, cervical, and rectal cancers (Fig. 2). The subcohort groups comprised 1100 and 4400 patients with and without cancer, respectively.

**Table 1:** Characteristics of the Study Subjects

	Non-cancer	Cancer	*p*-value
	*N* = 8784	*N* = 2196	
Age	71.09 ± 7.19	71.4 ± 7.09	0.0739
Age Group			0.9160
20–39	9 (0.10)	2 (0.09)	
40–59	593 (6.75)	140 (6.38)	
60–79	7339 (83.55)	1847 (84.11)	
> =80	843 (9.60)	207 (9.43)	
Gender			1.0000
Female	6100 (69.44)	1525 (69.44)	
Male	2684 (30.56)	671 (30.56)	
CCI			0.8847
0	1457 (16.59)	355 (16.17)	
1	2158 (24.57)	556 (25.32)	
2	2105 (23.96)	520 (23.68)	
2+	3064 (34.88)	765 (34.84)	
Comorbidities			
Myocardial infarct	198 (2.25)	59 (2.69)	0.2304
congestive heart failure	980 (11.16)	254 (11.57)	0.5865
peripheral vascular	294 (3.35)	73 (3.32)	0.9577
cerebrovascular disease	1796 (20.45)	458 (20.86)	0.6706
dementia	231 (2.63)	63 (2.87)	0.5348
chronic lung disease	3497 (39.81)	869 (39.57)	0.8378
connective tissue disease	658 (7.49)	149 (6.79)	0.2569
Ulcer	4583 (52.17)	1134 (51.64)	0.6535
chronic liver disease	1407 (16.02)	339 (15.44)	0.5058
Diabetes	2455 (27.95)	617 (28.1)	0.8901
diabetes with end organ damage	367 (4.18)	102 (4.64)	0.3333
Hemiplegia	92 (1.05)	22 (1)	0.8506
moderate or severe kidney disease	642 (7.31)	178 (8.11)	0.2039
Moderate or severe liver disease	–	–	
AIDS	–	–	
Infection	164 (1.87)	38 (1.73)	0.6700
1-year Mortality	146 (1.66)	90 (4.10)	<.0001
Chemotherapy	0 (0.00)	708 (32.24)	
Metastasis	0 (0.00)	7 (0.32)

### One-year infection rate of prosthetic joints

The number of patients with infected prosthetic joints 1 year after TKA were 38 (1.73%) and 164 (1.87%) in the cancer and control groups, respectively (Table 1). A multivariate-adjusted model revealed no association between infection and cancer (Table 2). In the unadjusted analysis, diabetes with end organ damage was associated with prosthetic joint infection (sHR, 1.77; 95% CI, 1.03–3.06); however, this association was nonsignificant in the Fine and Gray regression model (Table 2). The status of metastatic disease but not the use of chemotherapy was associated with prosthetic joint infection (Table 2 and Table 3).

**Table 2:** Prediction for infection

	Crude		Adjusted	
	sHR^a^(95%C.I.)	*p*-value	sHR^a^(95%C.I.)	*p*-value
Cancer vs. Non-cancer	0.92 (0.64–1.32)	0.6374	0.91 (0.63–1.31)	0.6089
Comorbidities				
Myocardial infarct	1.58 (0.74–3.36)	0.2355	1.47 (0.68–3.18)	0.3334
congestive heart failure	1.18 (0.77–1.79)	0.4473	1.07 (0.70–1.64)	0.7466
peripheral vascular	1.42 (0.73–2.78)	0.3036	1.33 (0.68–2.63)	0.4084
cerebrovascular disease	1.22 (0.87–1.69)	0.2500	1.08 (0.76–1.53)	0.6857
dementia	1.79 (0.92–3.51)	0.0874	1.63 (0.82–3.24)	0.1631
chronic lung disease	1.17 (0.88–1.55)	0.2827	1.11 (0.82–1.50)	0.4975
connective tissue disease	1.06 (0.63–1.80)	0.8218	1.05 (0.62–1.78)	0.8579
Ulcer	1.10 (0.83–1.46)	0.5153	1.03 (0.77–1.38)	0.8437
chronic liver disease	1.09 (0.75–1.59)	0.6491	1.07 (0.73–1.57)	0.7438
Diabetes	1.14 (0.84–1.54)	0.4175	0.99 (0.71–1.40)	0.9698
diabetes with end organ damage	1.77 (1.03–3.06)	0.0401	1.67 (0.91–3.06)	0.1007
Hemiplegia	1.52 (0.48–4.80)	0.4733	1.28 (0.40–4.12)	0.6779
moderate or severe kidney disease	1.12 (0.67–1.87)	0.6631	1.07 (0.64–1.79)	0.7902
Moderate or severe liver disease	–		–	
AIDS	.-		–	
Chemotherapy	0.96 (0.54–1.72)	0.8913	0.99 (0.47–2.09)	0.9815
Metastasis	8.66 (1.24–60.50)	0.0296	10.13 (1.19–86.34)	0.0343

sHR^a^: subdistribution hazard ratio

**Table 3:** Prediction for infection(only patients with Cancer)

	Adjusted	
	sHR^a^(95%C.I.)	*p*-value
Comorbidities		
Myocardial infarct	–	
congestive heart failure	1.21 (0.45–3.26)	0.7122
peripheral vascular	1.58 (0.36–7.02)	0.5493
cerebrovascular disease	1.65 (0.78–3.48)	0.1866
dementia	2.81 (0.80–9.85)	0.1060
chronic lung disease	0.76 (0.37–1.56)	0.4507
connective tissue disease	1.80 (0.63–5.20)	0.2757
Ulcer	1.59 (0.81–3.12)	0.1759
chronic liver disease	1.12 (0.46–2.72)	0.7990
Diabetes	0.78 (0.33–1.81)	0.5613
diabetes with end organ damage	1.87 (0.47–7.52)	0.3770
Hemiplegia	1.92 (0.24–15.21)	0.5363
moderate or severe kidney disease	0.94 (0.28–3.14)	0.9147
Moderate or severe liver disease		
AIDS		
Chemotherapy	1.02 (0.49–2.14)	0.9608
Metastasis	11.10 (1.22–101.30)	0.0328

sHR^a^: subdistribution hazard ratio

### Mortality rates in cancer patients after TKA

**Fig. 3:**
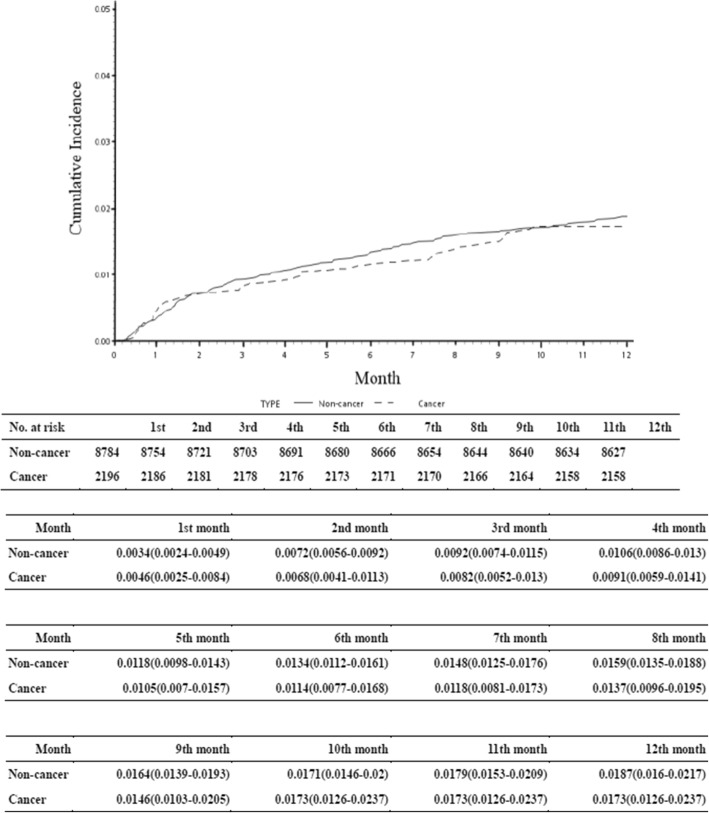
1-year mortality rate after total knee arthroplasties

The 1-year mortality rate after TKA was significantly higher (*p* < 0.001) in the cancer group (90 patients; 4.10%), (1-year cumulative incidence of 1.73%; 95% confidence interval [CI], 1.26–2.37%) than in the control group (146 patients; 1.66%), (1-year cumulative incidence of 1.71%; 95% CI, 1.46–2.0%) (Tables 1, 4 and Fig. 3). The 1-year relative survival rate was 97.52%. The Fine and Gray regression model did not show a significant association between metastasis and postoperative mortality (Table 4).

**Table 4:** Prediction for mortality

	Adjusted	
	sHR^a^(95%C.I.)	*p*-value
Cancer vs. Non-cancer	2.46 (1.71–3.52)	<.0001
Comorbidities		
Myocardial infarct	1.98 (0.98–3.99)	0.0557
congestive heart failure	1.56 (0.98–2.49)	0.0632
peripheral vascular	0.53 (0.17–1.64)	0.2717
cerebrovascular disease	0.83 (0.57–1.20)	0.3263
dementia	0.97 (0.40–2.34)	0.9477
chronic lung disease	1.26 (0.74–2.17)	0.3943
connective tissue disease	1.23 (0.65–2.32)	0.5240
Ulcer	1.31 (0.67–2.56)	0.4305
chronic liver disease	1.43 (0.32–6.48)	0.6403
Diabetes	1.04 (0.67–1.63)	0.8608
diabetes with end organ damage	1.24 (0.55–2.79)	0.5993
Hemiplegia	1.80 (0.52–6.24)	0.3566
moderate or severe kidney disease	1.84 (0.64–5.32)	0.2614
Moderate or severe liver disease	–	
AIDS	–	
Metastasis	1.17 (0.68–2.01)	0.5754
	–	

sHR^a^: subdistribution hazard ratio

**Fig. 4:**
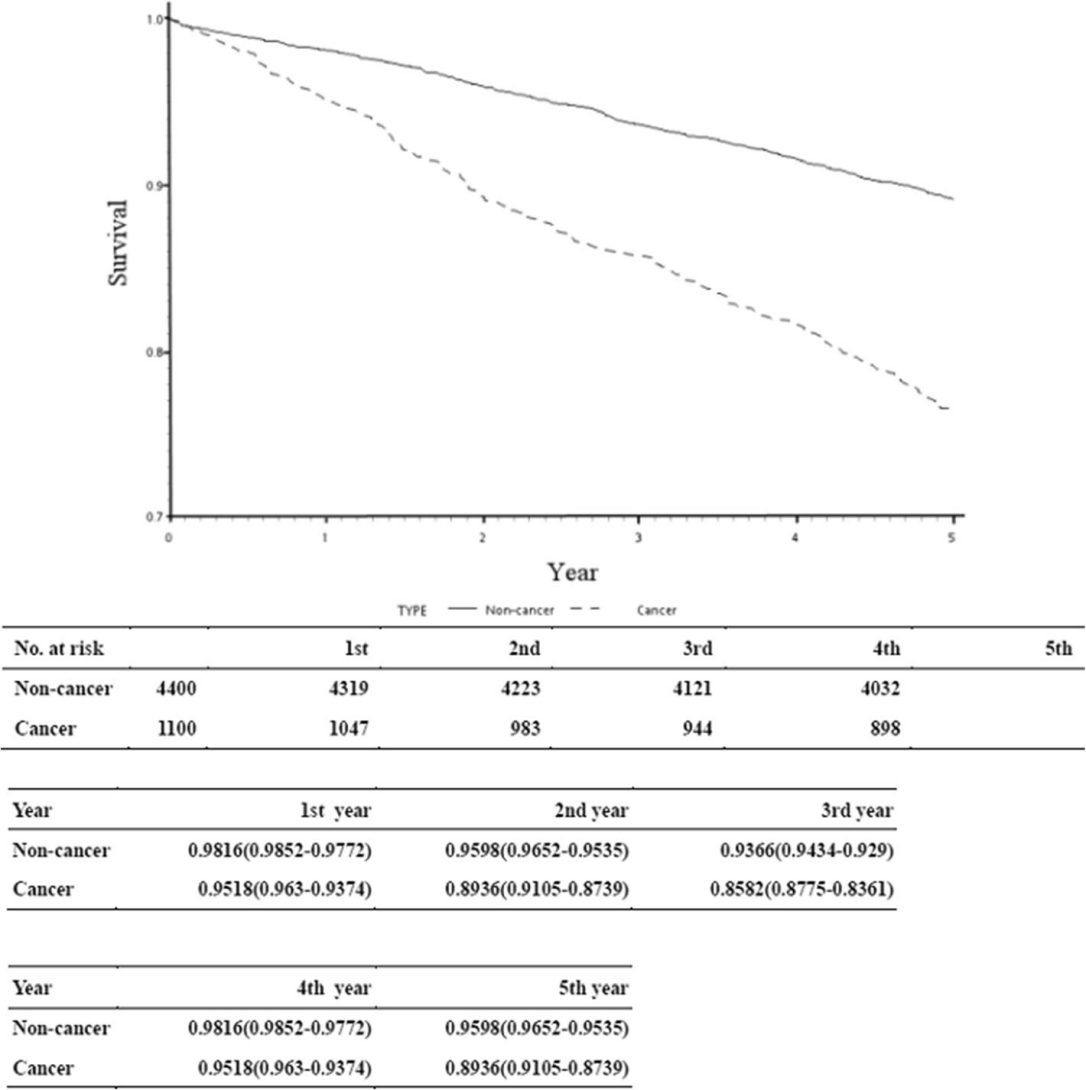
5-years overall survival rate of cancer patients after total knee arthroplasties

In the 5-year follow-up, a significantly lower overall survival rate was observed in cancer patients as compared with the controls (Fig. 4). The overall 5-year survival rate was 89.36% in the cancer cohort and was relatively 93.10% as compared with the non-cancer controls.

## Discussion

Infection is common in patients with cancer [20], because several risk factors—such as neutropenia [21], cellular immune dysfunctions (e.g., defects in T-lymphocytes and mononuclear phagocytes), humoral immune dysfunction, bone marrow and stem cell transplantation, local factors (e.g., tumor metastasis and operative procedures), use of central venous catheters, splenectomy, and use of chemotherapeutic agents [22], lower their resistance to infections [23]. Gram-positive organisms cause approximately 50%–55% of all infectious diseases in cancer patients with neutropenia, and Staphylococci are the most common organisms isolated from neutropenic and nonneutropenic cancer patients [24]. Although no studies have reported periprosthetic infection rates in cancer patients after TKA, some studies have reported an increase in deep infection rates of up to 9.1% after TKA in immunocompromised patients such as patients with AIDS [25].

Under these detrimental defence circumstances, one can reasonably suspect higher infection rates in cancer patients after TKA. However, this study revealed that the 1-year periprosthetic infection rate of 1.73% in cancer patients is not significantly higher than that (1.87%) in non-cancer patients. The result showed that the use of chemotherapy did not increase the risk of infection. Although this study does not provide information on prophylactic strategies for periprosthetic knee joint infections, our results suggest that the currently used prophylactic methods are effective for cancer patients who have undergone TKA. Nevertheless, orthopedicians must pay attention to the immunocompromised conditions especially in neutropenic status caused by chemotherapy in cancer patients receiving TKA in order to prevent periprosthetic knee joint infections.

A decrease in the long-term survival rate of patients receiving TKA most likely reflects the natural process of aging [25]. However, a higher mortality rate than usual is expected when cancer patients receive TKA, possibly because of a trend similar to that in the United States, where cancer is the leading cause of death in people aged less than 85 years [26]. The post-TKA 1-year mortality rate (4.10%) was significantly higher in cancer patients in the present study. But metastasis diseases are not associated with mortality in our study. We think that this phenomenon is caused by lower desire of received TKA in cancer patients with metastatic disease. Nevertheless, because of advances in the control, prevention, early detection, and treatment of cancer since 1990 [27, 28], cancer-related death rates have decreased. Cancer mortality rates have declined by approximately 1% annually and by more than 25% in the last 25 years [29]. The 5-year relative survival rate was 93.10% in the present study; the majority of the patients had ample time to experience the benefits of TKA, including functional improvement of the knee, knee joint pain relief, and improved quality of life.

Our study has some limitations that should be addressed. First, the severity of the comorbidities could not be determined from the NHIRD. Second, data on cancer staging was unavailable, which might induce a healthy patient bias. The numbers of metastatic diseases were small in our study. We are unable to investigate the real effect of metastatic diseases on the mortality rate. Nevertheless, rather than create a spurious association, such a stringent inclusion criteria would bias the results toward a null association. Finally, the effect of unaccounted confounders cannot be ruled out; for example, we could not examine the potential influence of body weight, cigarette smoking, alcohol drinking, and dietary habits because this information is unavailable in the NHIRD. Moreover, because the data is deidentified, we could not collect this information from the patients directly. The merits of this study are that the NHIRD is representative of all residents of Taiwan and that there was no loss to follow-up.

## Conclusion

In conclusion, after TKA, similar 1-year periprosthetic infection rates but differing 1-year mortality rates were observed in patients with and without cancer. The high 5-year relative survival rates in cancer patients who underwent TKA indicate that TKA is a feasible treatment option for cancer patients with severe OA.

## Appendix

**Table 5:** ICD-9-CM codes and the corresponding diseases or procedures

Disease or procedures	Corresponding ICD-9-CM codes
all cancer	140.XX-208.XX
	Exclusion: musculoskeletal cancers 170.0–170.9
Metastasis	164.8; 165.8
	196.0~ 196.9
	197.0~ 197.8
	198.0~ 198.7
	198.81; 198.82; 198.89
Chemotherapy management	05221A; 37005B; 37025B
	37031B~37041B
Total knee replacement	64164B
Periprosthetic Infection of Knee joint	
infection due to internal device	996.66; 996.67; 996.69
surgical wound infection	998.5; 998.51; 998.59
osteomyelitis	730.01~ 730.04; 730.07
	730.11~ 730.14; 730.17
	730.21~ 730.24; 730.27
	730.31~ 730.34; 730.37
	730.7; 730.70~ 730.79
	730.81~ 730.84; 730.87
	730.91~ 730.94; 730.97
Septic arthritis	711.01~ 711.04; 711.07
	711.1; 711.10~ 711.19
	711.2; 711.20~ 711.29
	711.31~ 711.34; 711.37
	711.41~ 711.44; 711.47
	711.51~ 711.54; 711.57
	711.61~ 711.64; 711.67
	711.7; 711.70~ 711.79
	711.8; 711.81~ 711.89
	711.91~ 711.94; 711.97
Surgical intervention for periprosthetic infection of knee joint	64004C, 64005B, 64053B, 64055B,64198B, 48004C, 48005C, 48006C; Exclusion: 64003C, 64056B, 64057B, 64200B, 64199B

Footnotes: ICD-9-CM, International Classification of Diseases, 9th Revision, Clinical Modification;

## Abbreviations

HR: Hazard ratio
NHI: National Health Insurance
NHIRD: Taiwan National Health Insurance Research Database
OA: Osteoarthritis
TKA: Total knee arthroplasty
